# Influence of Customer Quality Perception on the Effectiveness of Commercial Stimuli for Electronic Products

**DOI:** 10.3389/fpsyg.2016.00336

**Published:** 2016-03-14

**Authors:** Álvaro Garrido-Morgado, Óscar González-Benito, Mercedes Martos-Partal

**Affiliations:** Departamento de Administración y Economía de la Empresa, Universidad de SalamancaSalamanca, Spain

**Keywords:** quality perceptions, customer loyalty, commercial stimuli, displays, advertising flyers, cue utilization theory, consumer information processing, time theory

## Abstract

Creating and maintaining customer loyalty are strategic requirements for modern business. In the current competitive context, product quality, and brand experience are crucial in building and maintaining customer loyalty. Consumer loyalty, which may be classified into cognitive loyalty and affective loyalty, is related to customers' quality perception. Cue utilization theory distinguishes two dimensions for perceived quality, extrinsic quality—linked to the brand—and intrinsic quality—related with internal product characteristics. We propose that (i) cognitive loyalty is more influenced by intrinsic product quality whereas extrinsic product quality (brand name) is more salient for affective loyalty and, (ii) different commercial stimuli have a differential effectiveness on intrinsic and extrinsic perceived quality. In fact, in this study, we analyze how perceived quality dimensions may influence the effectiveness of two different commercial stimuli: displays and advertising flyers. While displays work within the point of sale under time-constrained conditions where consumers are more likely to use heuristics to simplify their decisions, advertising flyers work outside of the point of sale under low time-constrained conditions, and therefore favor a more reasoned purchase decision where systematic processing will be more likely. We analyze the role of quality perception in determining the effectiveness of both these commercial stimuli for selling products that induce high purchase involvement and perceived risk. The empirical analysis focuses on computer products sold by one of Europe's largest computer retailers and it combines scanner, observational, and survey data. The results show that both dimensions of quality perceptions moderate the influence of displays and advertising flyers on sales, but their impact is different on each commercial stimuli. Extrinsic quality perception increases to a greater extent the effect of displays due to the use of a brand name heuristic. However, intrinsic quality perception improves to a greater extent the effect of advertising flyers, which in turn are more closely related to systematic decision processing.

## Introduction

Today the current economic situation is reflected in the slowdown in household consumption expenditure in Europe in recent years. In fact, some Euro area countries, such as France, Germany, Italy or Spain, have presented negative year-on-year average rates in 2013 or 2014 (The World Bank, 2015). Therefore, the economic situation causes increasingly more competition for both retailers and manufacturers that are struggling to attract consumers to their stores and products (Lin et al., 2013). Because of this, retailers and manufacturers try to analyze consumers' needs and preferences in order to be able to adapt the offer to increase customer loyalty to their stores/brands. Therefore, they adjust their prices, improve their product quality and increase their communication effort to make their new offers more noticeable in order to improve their sales (Cant and Hefer, 2014).

In fact, retailers use different commercial stimuli that take place both inside and outside the store. On one hand, in-store stimuli, such as merchandising tools or special displays, are noticeable by customers inside the store at the moment in which they carry out their purchase decision (Bava et al., 2009; Bezawada et al., 2009; Phillips et al., 2015). Therefore, in-store stimuli trigger unrecognized needs and desires or trigger memories for forgotten needs, leading to in-store decision making, or unplanned purchasing (Inman et al., 2009), such as the purchase of one particular substitutive product or one particular brand instead of another. These stimuli usually trigger unplanned purchases, which now represent about 70% of total purchases (Stilley et al., 2010; Bell et al., 2011). On the other hand, out-of-store commercial stimuli, such as advertising flyers, are mainly used by retailers to increase traffic to the store or to publicize other promotions (Gijsbrechts et al., 2003; Schmidt and Bjerre, 2003; Ziliani and Ieva, 2015). Advertising flyers are sent to potential consumers' homes, facilitating their purchase planning (Schmidt and Bjerre, 2003), as they may carry out a more reasoned purchase decision under low time-constrained conditions.

The academic literature on commercial stimuli effectiveness has analyzed the moderating role of specific attributes of the stimuli on sales (e.g., redemption time in coupon effectiveness or placement of the display or number of pages and size of the advertising flyers; e.g., Bawa, 1996; Gijsbrechts et al., 2003; Bezawada et al., 2009; Barat and Ye, 2012; Luceri et al., 2014) or the effect of more general characteristics, such as consumer characteristics (e.g., price sensitiveness, value consciousness, coupon properness, variety seeking, impulsive personality, or planned and unplanned consumer; e.g., Bawa, 1996; Laroche et al., 2003; Wakefield and Inman, 2003; Swaminathan and Bawa, 2005; Govindasamy et al., 2007; Haans and Gijsbrechts, 2011; Lin et al., 2013; Gázquez-Abad et al., 2014; Gázquez-Abad and Martínez-López, 2016). Moreover, previous studies have shown the moderating role of product and brand characteristics (storage and perishable conditions, impulse purchase, hedonic/utilitarian nature, interpurchase cycle, brand tiers) on the effectiveness of commercial stimuli (e.g., Narasimhan et al., 1996; Lemon and Nowlis, 2002; Pauwels et al., 2002; Wakefield and Inman, 2003; Inman et al., 2009; Castro et al., 2013).

One very important product characteristic is customers' perceived product quality, as it is a key determinant in building and maintaining customer loyalty (Brakus et al., 2009; Pan et al., 2012; Akdeniz et al., 2014). Despite this, the research literature on the influence of perceived quality on commercial stimuli is limited. Furthermore, previous studies such as Lemon and Nowlis (2002) use price tiers as proxy of perceived quality and do not distinguish between different quality attributes. Product quality comprises two types of attributes: extrinsic and intrinsic attributes (Bava et al., 2009; Gooner and Nadler, 2012; Akdeniz et al., 2014). Whereas, extrinsic attributes (e.g., brand name) are more related to affective loyalty (customers build affect toward the brand on the basis of cumulatively satisfying usage occasions), intrinsic attributes have a more objective nature (e.g., the technical characteristics of electronic products), which can be compared easily by seeking out information about the product (Szibillo and Jacoby, 1974; Richardson et al., 1994) and, therefore, are more related to cognitive loyalty. Consequently, in this paper we attempt to fill in this gap by analyzing whether perceived product quality (extrinsic and intrinsic attributes) has a moderating role in the effectiveness of different commercial stimuli (product displays and advertising flyers).

The objective of this paper is to extend the research on commercial stimuli effects on sales by (i) examining and comparing the direct effect of one in-store stimuli (special displays) and one out-store stimuli (advertising flyers) on the sales of infrequently purchased products with high perceived risk and high involvement and (ii) analyzing the moderating role of perceived quality (intrinsic and extrinsic attributes) on the effectiveness of both commercial stimuli on sales.

Our paper contributes to the marketing, retail and consumer behavior literature by providing a theoretical framework based on cue utilization theory and consumer processing information and by making an empirical analysis to ascertain the differences in effectiveness of commercial stimuli regarding both dimensions of perceived quality on sales of technological products. Specifically our paper contributes to previous research in four ways.

First, we consider the two dimensions of perceived quality, intrinsic (product characteristic) and extrinsic (brand). Previous research has not analyzed the moderating role of intrinsic perceived quality and brand perceived quality on the effectiveness of commercial stimuli. The present study seeks to extend and improve the insights of the few previous studies that have attempted to capture the quality effect on effective commercial stimuli. For example, Lemon and Nowlis (2002) use price tiers as proxy of perceived quality of the brand. Liu-Thompkins and Tam (2013) have researched the differential effectiveness of cross-selling promotion on attitudinal loyal customer and spurious loyalty, but they ignore the differences between intrinsic perceived quality-cognitive loyalty and extrinsic perceived quality-affective loyalty which may undermine the effectiveness of commercial stimuli. Therefore, this study aims to analyze the effectiveness of different commercial stimuli depending on the intrinsic perceived quality of product and brand.

Second, we combine three data sources in our analysis: scanner data and observational data and customer surveys to conduct the empirical analyses. Previous studies assume the brand quality or use price tiers as proxy of perceived quality and do not distinguish between different quality attributes (e.g., Lemon and Nowlis, 2002). By contrast, this study obtains customer perceived quality by collecting a consumer survey (extrinsic perceived quality) or analyzing the product characteristics (intrinsic perceived quality).

Third, we study a high involvement product. Most prior studies about the effectiveness of commercial stimuli analyze frequently purchased products with low perceived risk and low involvement (e.g., Burton et al., 1999; Lemon and Nowlis, 2002; Gijsbrechts et al., 2003; Van Heerde et al., 2004; Bezawada et al., 2009). We study a high involvement product to generalize previous findings about the effect of commercial stimuli on frequently purchased products or detecting different effects due to differences in this type of product, such as the greater effect of perceived quality (extrinsic and intrinsic) compared to price. In this paper we study infrequently purchased products (computers), which involve high customer perceived risk and involvement. Previous studies on sales of technological products have analyzed the direct effect of the brand and objective quality on product sales (Neelamegham and Chintagunta, 2004; Sriram et al., 2006); however, the effect of different commercial stimuli have not been previously analyzed. Identifying the drivers of customer loyalty can help us attain a better understanding of consumer behavior and also allow resources to be allocated among marketing tactics.

Four, we analyze the effect of two different commercial stimuli, one in-store stimulus (product displays which represent a time-constrained condition) and one out-of-store stimulus (advertising flyers which represent a low time-constrained condition) on the sale of computer products. Some studies have used different tools at the same time (Narasimhan et al., 1996; Van Heerde et al., 2004; Ailawadi et al., 2006; Haans and Gijsbrechts, 2011); however, most of them use these commercial stimuli as control variables when they analyze promotion effectiveness on frequently purchased products and, therefore, do not compare and explain their effect on product sales depending on the customer perceived quality as we do in this study.

The next section offers a conceptual framework that leads directly into the study hypotheses. After a description of the methodology, we provide details on the empirical analysis and results, along with their interpretations. The final section summarizes the conclusions and implications of the present study.

## Conceptual framework: Proposed hypotheses

### Customer perceived quality

Quality is a central element in business strategy and academic research. Firms compete on quality, customers search for quality, and markets are transformed by quality (Golder et al., 2012). In the marketing literature, some researchers distinguish between objective and subjective quality (e.g., Mitra and Golder, 2006) whereas others, such as Zeithaml (1988), notes that “objective quality may not exist because all quality is perceived by someone.” In this paper we focus on customer perceived quality.

Perceived quality is defined as a buyer's estimate of a product's cumulative excellence (Zeithaml, 1988). Perceived product quality is a key variable in the consumer decision process (Steenkamp, 1990) and it is considered a pivotal determinant of shopping behavior and product choice (e.g., Zeithaml, 1988; Grewal et al., 1998; Wang, 2013; Akdeniz et al., 2014).

According to information economics (Nelson, 1970, 1974), consumers have uncertainty about the quality attributes and benefits of the products they aim to purchase because of the imperfect, asymmetric information that characterizes most product markets. Companies are more informed about their products than customers, so firms can behave opportunistically. To overcome that uncertainty, companies must inform consumers and give them cues about their credibility (Erdem and Swait, 1998; Akdeniz et al., 2014). Cue signals mostly serve as heuristics in assessing product quality when (1) there is a need to reduce the perceived risk of purchase, (2) the consumer lacks expertise and consequently the ability to assess quality, (3) consumer involvement is low, (4) objective quality is too complex to assess or the consumer is not in the habit of spending time objectively assessing quality, or (5) there is an information search preference and need for information (Dawar and Parker, 1994). Therefore, in technological products (e.g., computers) the use of cues will be very useful to simplify customer decision process.

Cue utilization theory considers that products consist of a set of cues that serve as surrogate indicators of quality to consumers and specifies that, when consumers make inferences about perceptions (Olson, 1978). Cues are represented by the set of attributes related to the product they are assessing. The particular cues are evoked according to their predictive and confidence values. The predictive value of a cue is the degree to which consumers associate a given cue with product quality. The confidence value of a cue is the degree to which consumers have confidence in their ability to use and judge that cue accurately (Richardson et al., 1994).

These cues can be either extrinsic or intrinsic. The former relate less closely to the product, such that changes to the extrinsic cue do not necessarily entail changes in product attributes (e.g., brand names, packaging, and product communication). The latter include attributes whose modification would involve a change in the physical properties of the product (e.g., ingredients in food products or technical characteristics in computers). Research evidence suggests that consumers tend to use both extrinsic and intrinsic cues concurrently when evaluating product quality (Szibillo and Jacoby, 1974; Richardson et al., 1994; Gooner and Nadler, 2012; Akdeniz et al., 2014).

Dawar and Parker (1994) propose that the relative importance of these cue signals generally follow their specificity, or the extent to which a particular signal is not shared across competitive products. A brand name, for example, is typically shared by only a few products within a competitive line of products and is therefore a very specific signal. Physical features, on the other hand, can be shared to a greater extent across competing products and are therefore less specific. The more specific a signal, all else being equal, the more likely it will provide information that is useful in an assessment of product quality. This distinction is consistent with the belief that cue signals are relied on as a function of their predictive value.

Consumers use cues to develop beliefs about products and that task response (i.e., choice or evaluation) may be a direct function of these mediating beliefs (Olson, 1978; Dawar and Parker, 1994; Richardson et al., 1994; Aqueveque, 2006, 2008; Akdeniz et al., 2014).

### Customer brand loyalty

Customer loyalty remains a topic of great interest for firms (Kotler and Keller, 2009; Garnefeld et al., 2013; Wieseke et al., 2014). Consumers exhibit behavioral loyalty when they repeatedly patronize a business, often to the exclusion of competing offers. Behavioral loyalty is desirable from a financial perspective, for example, because superior brand performance outcomes such as greater market share and price premiums relate to customer brand loyalty (Chaudhuri and Holbrook, 2001).

In a comprehensive discussion of customer brand loyalty, Oliver (1999) defines loyalty as “a deeply held commitment to rebuy or repatronize a preferred product/service consistently in the future, thereby causing repetitive same-brand or same brand-set purchasing, despite situational influences and marketing efforts having the potential to cause switching behavior.” According to his framework, attitudinal loyalty addresses the psychological component of a consumer's commitment to a brand and may encompass beliefs of product/service superiority as well as positive and accessible reactions toward the brand. This definition emphasizes the two different aspects of brand loyalty: behavioral and attitudinal (Dick and Basu, 1994; Chaudhuri and Holbrook, 2001; Garnefeld et al., 2013).

Oliver's (1999) framework proposes a cognition-affect-conation-action framework with four loyalty phases. In the first loyalty phase, the brand attribute information available to the consumer indicates that one brand is preferable to its alternatives. This stage is referred to as cognitive loyalty, or loyalty based on brand belief only. Cognition can be based on prior knowledge or on recent experience-based information. Loyalty at this phase is directed toward the brand because of this information (attribute performance levels).

In the second phase of loyalty development, a liking or attitude toward the brand has been developed on the basis of cumulatively satisfying usage occasions. Commitment at this phase is referred to as affective loyalty and is encoded in the consumer's mind as cognition and affect.

The next phase of loyalty development is the conative (behavioral intention) stage, influenced by repeated episodes of positive affect toward the brand. Conation, by definition, implies a brand-specific commitment to repurchase.

In the action control sequence, the motivated intention in the previous loyalty state is transformed into readiness to act. The action control paradigm proposes that this is accompanied by an additional desire to overcome obstacles that might prevent the act. Action is perceived as a necessary result of engaging both these states. If this engagement is repeated, an action inertia develops, thereby facilitating repurchase.

In short, cognitive loyalty focuses on the brand's performance aspects, affective loyalty is directed toward the brand's likeableness, conative loyalty is experienced when the consumer focuses on wanting to rebuy the brand, and action loyalty is commitment to the action of rebuying (Oliver, 1999). Dick and Basu (1994) refer to behavioral loyalty without attitudinal loyalty as “spurious loyalty.”

In the current competitive context product quality and brand experience are crucial in building and maintaining customer loyalty (Brakus et al., 2009; Pan et al., 2012; Schmitt et al., 2014; Wieseke et al., 2014; Lin et al., 2015).

Brand experience is defined as subjective, internal consumer responses (sensations, feelings, and cognitions) and behavioral responses evoked by brand-related stimuli that are part of a brand's design and identity, packaging, communications, and environments (Brakus et al., 2009). If a brand evokes an experience, this alone leads to loyalty. In addition, an experience may be the basis for more elaborative information processing and inference making that results in brand-related associations. In turn, these associations affect loyalty (Brakus et al., 2009).

In this paper, we focus on cognitive and affective loyalty. We propose that in the first loyalty phase (cognitive loyalty) the customer is more focused on utilitarian and performance product attributes, therefore intrinsic quality will be a more relevant signal in this phase. However, in the second phase (affective loyalty), because affect toward the brand has been developed on the basis of cumulatively satisfying experience occasions, the customer can be driven by the positive reaction toward a brand signal (extrinsic quality).

### Commercial stimuli

Commercial stimuli are informative cues (Steenkamp, 1990) that aim to attract attention to and raise interest in a product, by featuring it inside the store (special location or displays) or outside the store (coupons and advertising flyers; e.g., Bawa, 1996; Gijsbrechts et al., 2003; Bezawada et al., 2009; Inman et al., 2009; Bava et al., 2009; Haans and Gijsbrechts, 2011; Luceri et al., 2014). These stimuli trigger a cognitive or emotional response to the featured product and, therefore, can become part of the set of considered options in the evaluation phase or choice, by enhancing its purchase probability (Yeung and Wyer, 2004; Chandon et al., 2009; Inman et al., 2009). This paper focus on two of the most used commercial stimuli: displays (as in-store stimuli) and advertising flyers (as out-of-store stimuli).

On one hand, in-store stimuli (special displays) can be considered as any special presentation in the store aimed at drawing attention to a product and increasing its sales (e.g., islands, end-of-aisle displays, shelf signages, etc.; Bezawada et al., 2009; Garrido-Morgado and González-Benito, 2015; Phillips et al., 2015). These special displays are noticeable to customers inside the store, i.e., at the moment in which they decide and carry out their purchase decision (Bava et al., 2009; Bezawada et al., 2009; Inman et al., 2009). Therefore, displays may trigger unrecognized needs and desires or trigger memories for forgotten needs, leading to in-store decision making, or unplanned purchasing (Inman et al., 2009). What is more, consumers tend to view them as special bargains and often buy from a displayed product, which they had no previous intention of buying (Lin et al., 2013). Thus, they are usually related to unplanned purchases, which now represent around 70% of total purchases (Stilley et al., 2010; Bell et al., 2011).

Several studies confirm that displays or merchandising techniques exert direct effects on sales (e.g., Lemon and Nowlis, 2002; Chandon et al., 2009; Bezawada et al., 2009; Inman et al., 2009), usually by adopting displays as a control variable in their analyses of the effects of promotions (e.g., Narasimhan et al., 1996; Van Heerde et al., 2004).

On the other hand, out-of-store stimuli (advertising flyers) are mass communication techniques that are mainly used by retailers to increase the store traffic as well as to publicize promotions or to increase purchases (Burton et al., 1999; Haans and Gijsbrechts, 2011; Luceri et al., 2014). Advertising flyers are sent to potential consumers' homes in order to remind consumers about the existence of a product or to inform them about any promotions to enhance the impact of those deals (Gijsbrechts et al., 2003; Schmidt and Bjerre, 2003; Luceri et al., 2014). Thus, advertising flyers work differently from displays because they may facilitate the purchase planning (Burton et al., 1999; Govindasamy et al., 2007).

Previous studies confirm that advertising flyers, in particular, exert direct effects on store traffic and store sales as well as product sales (Burton et al., 1999; Gijsbrechts et al., 2003; Schmidt and Bjerre, 2003; Haans and Gijsbrechts, 2011; Luceri et al., 2014). However, most of the previous literature tends to include advertising flyers in a “feature” control variable that refers to any external technique designed to increase sales of a product or attract consumers to the store when they focus on other issues such as promotions (Narasimhan et al., 1996; Van Heerde et al., 2004; Ailawadi et al., 2006).

Based on this previous literature, we expect a positive effect of both commercial stimuli, displays and advertising flyers, on sales. Therefore, we propose the following hypotheses:

H1: Product displays engender a positive effect on sales
H2: Advertising flyers engender a positive effect on sales

### The moderating role of perceived product quality on commercial stimuli effectiveness

Studies about commercial stimuli usually take into account different typical characteristics of the analyzed stimuli and thus control for various particular aspects that can moderate their results (see Table 1). For example, studies that analyze special presentations at the point of sale control for the distance between the new position of the displayed product from its usual position or whether this new position is in proximity to a complementary product or another commercial stimulus (Bezawada et al., 2009; Chandon et al., 2009; Phillips et al., 2015). Studies focused on advertising flyers also account for their characteristics, such as the number of pages, geographic area in which they are launched, temporal frequency, or average discounts (Gijsbrechts et al., 2003; Luceri et al., 2014). Studies about promotions frequently control for their characteristics, such as whether they induce immediate benefits, for example a price discount, a gift, or additional amounts of the stimulated product (Hardesty and Bearden, 2003; Palazón and Delgado-Ballester, 2009; Yi and Yoo, 2011).

**Table 1:** **Summary of previous studies about commercial stimuli**.

**Study**	**Analyzed commercial stimuli**			**Moderating factors that control stimuli effectiveness**		
	**Promotion**	**Display**	**Feature**	**Stimuli characteristic**	**Consumer characteristic**	**Product characteristic**
Ailawadi et al., 2006^a^	✓		✓			✓
Barat and Ye, 2012			✓	✓		
Bava et al., 2009		✓			✓	
Bawa, 1996			✓	✓	✓	✓
Bezawada et al., 2009		✓		✓		✓
Burton et al., 1999			✓		✓	
Cant and Hefer, 2014		✓		✓	✓	
Castro et al., 2013		✓				✓
Chandon et al., 2009		✓		✓	✓	✓
Garrido-Morgado and González-Benito, 2015	✓	✓		✓		✓
Gázquez-Abad and Martínez-López, 2016			✓		✓	
Gázquez-Abad et al., 2014			✓		✓	
Gijsbrechts et al., 2003			✓	✓		
Govindasamy et al., 2007			✓		✓	
Haans and Gijsbrechts, 2011	✓	✓	✓	✓	✓	
Hardesty and Bearden, 2003	✓			✓		
Inman et al., 2009		✓	✓		✓	✓
Laroche et al., 2003	✓		✓	✓	✓	
Lemon and Nowlis, 2002	✓	✓	✓			✓
Lin et al., 2013	✓				✓	✓
Luceri et al., 2014			✓	✓		✓
Narasimhan et al., 1996^a^	✓	✓	✓			✓
Nowlis and Simonson, 2000	✓					✓
Palazón and Delgado-Ballester, 2009	✓			✓		
Pauwels et al., 2002	✓					✓
Phillips et al., 2015		✓		✓		
Schmidt and Bjerre, 2003			✓		✓	
Swaminathan and Bawa, 2005			✓		✓	
Van Heerde et al., 2004^a^	✓	✓	✓	✓		
Wakefield and Inman, 2003	✓				✓	✓
Yi and Yoo, 2011	✓			✓		

a *This study focuses on promotions, but it adds display and feature as control variables*.

Furthermore, other studies analyze the key moderating role of consumers' profile on commercial stimuli effectiveness. For example, consumer characteristics such as coupon proneness, brand loyalty, store loyalty, value consciousness, price consciousness, or income (Bawa, 1996; Laroche et al., 2003; Wakefield and Inman, 2003; Swaminathan and Bawa, 2005) have an impact on the effectiveness of different promotions or stimuli.

Other important moderating issues of commercial stimuli effectiveness are product characteristics, such as its storage conditions, impulsive buying, hedonic/utilitarian benefit (Narasimhan et al., 1996; Wakefield and Inman, 2003), or brand characteristics (Nowlis and Simonson, 2000; Lemon and Nowlis, 2002). In this line, Lemon and Nowlis (2002) examine synergies between different types of promotions (price promotions, displays, and feature advertising) and characteristics of the brands that offer the promotions. They consider the price-quality tier of the brand as a moderator of the effectiveness of promotions. Specifically they consider leading national brands as the high quality tier and private labels or small share brands as the low-quality tiers by assuming price tiers as quality tiers. They found that high tier brands benefit more than low-tier brands from price promotions, display, or feature advertising when the promotional tools are used by themselves.

However, Lemon and Nowlis (2002), as in the majority of studies, performed their research on frequently purchased products and did not take into account that the product perceived quality is formed through both types of characteristics, product characteristics—intrinsic attributes—and brand characteristics—extrinsic attributes—(Szibillo and Jacoby, 1974; Richardson et al., 1994). Their results may be moderated if the study focuses on infrequently purchased products (e.g., computers). This product features substantial technological components and greater perceived risk and high involvement, because of their complexity and dynamic evolution (Laurent and Kapferer, 1985; Neelamegham and Chintagunta, 2004; Sriram et al., 2006). For these reasons, consumers may be motivated to develop a more reasoned and planned process for purchasing this type of product and, therefore, the degree to which alternatives can be compared directly or must be considered individually may become a very important aspect of the decision task that can affect a wide variety of decisions (Hsee and Leclerc, 1998; Ritov, 2000) as well as the effectiveness of different commercial stimuli depending on the degree to which they favor a more reasoned purchase and allow products to be directly compared or considered separately.

Previous research shows that time constraints influence consumers' perceptions of quality (Suri and Monroe, 2003). Dhar and Nowlis (1999) found that under time pressure, consumers are more likely to consider unique features among choices and less likely to consider common features. In addition, their subjects recalled more features (unique and common) in the no time pressure condition than in the time pressure condition. Under time-constrained conditions, customers are more likely to use heuristics, such as the brand name heuristic, to simplify the cognitive task (Kaplan et al., 1993; Nowlis, 1995). Whereas, in low time-constrained conditions the opportunity to process information is high and then systematic processing will be more likely (Suri and Monroe, 2003). Moreover, an increase in time pressure led to a greater use of heuristics when the motivation to process information was high relative to when it was low (Sanbonmatsu and Fazio, 1990; Suri and Monroe, 2003), for example with a high involvement product like computers.

Displays usually isolate a displayed product from the rest of competing alternatives. These stimuli take place within the store where consumers have less time to reason the purchase; therefore, they trigger a more unplanned purchase and are more likely to use heuristics to simplify decisions. Displays are used for highlighting a particular product and consumers consider this product individually. In this context, the brand is a unique feature among choices and brand name could be used as a heuristic by the consumer as a risk reduction strategy (Fischer et al., 2010; Gooner and Nadler, 2012). Brands identify the source or maker of a product. Consumers recognize a brand and activate their knowledge about it (Zhang and Sood, 2002). Using what they know about the brand in terms of overall quality and specific characteristics, consumers can form reasonable expectations about the functional and other benefits of the brand. Consequently, brands contribute to reducing the consumer's (subjective) risk of making a purchase mistake (e.g., Kapferer, 2008; Keller, 2008) and may be used for avoiding a more complicated purchase process. Moreover, in the case of display, brand is a more salient cue because it is a more specific signal which provides more information about the quality of the product (Dawar and Parker, 1994).

Advertising flyers are out-of-store stimuli because they are usually sent to potential consumers' homes in order to facilitate their purchase planning and, ideally, to attract them to the store (Schmidt and Bjerre, 2003; Haans and Gijsbrechts, 2011). Advertising flyers allow consumers to have time to overthink the purchase decision, giving them time to search for information and evaluate the perceived quality of a product in relation to all its attributes before they even enter the point of sale (Gijsbrechts et al., 2003). Although advertising flyers also encourage separate evaluations of products, in this context in low time-constrained conditions the consumers have more time to reason the purchase. Thus, they have the opportunity to process more information and therefore it is more likely that consumers will carry out more systematic information processing in which they analyze the intrinsic characteristics of the product. They may easily compare information about different product alternatives by using other available communication channels like the Internet.

Thus, we propose that the perceived quality attributed to the brand is more salient, and thus engenders greater sales, with displays than with advertising flyers. However, the perceived quality attributed to the intrinsic product characteristics is more salient, and thus engenders greater sales, with advertising flyers than with displays. Therefore, in line with this argument, we present the following hypotheses:

H3: The perceived quality attributed to the brand enhances the effectiveness of displays more than it does the effectiveness of advertising flyers for sales.
H4: The perceived quality attributed to the intrinsic product characteristics enhances the effectiveness of advertising flyers more than it does the effectiveness of displays for sales.

## Methods

### Study context

For this study, we analyzed computer products instead of the more frequently analyzed grocery products or FMCG (Fast-Moving Consumer Goods). We considered this type of product to be the most appropriate for analyzing the influence of the quality attributed to the brand—very often used as a risk reduction strategy for this product category—and the intrinsic product quality characteristics—very easy to compare through objective measures like *MegaBites* or *GigaBites*—on the effectiveness of different commercial stimuli.

Although consumers usually plan their purchase of computers rather than buy them on impulse, they usually have product experience and high motivation to process information (Suri and Monroe, 2003); therefore, commercial stimuli can trigger the purchase of one particular SKU (Stock-Keeping Unit) or one brand instead of another. Also, this type of infrequently purchased product with substantial technological components in dynamic evolution is more complex and, therefore, is usually linked to lower knowledge, greater perceived risk and higher involvement (Neelamegham and Chintagunta, 2004; Sriram et al., 2006).

In fact, consumers often have a purchase intention about the product category they need or want to buy, but they do not know exactly what particular SKU or brand to buy. In these situations, they may decide what SKU or brand to acquire by planning their purchase through the search for information about the product category before going into the store. A second option, especially for consumers without much knowledge about the product, is to decide the SKU or brand within the store by paying attention to the commercial stimuli and sell staff's recommendations or, even, to brand perceived quality in order to simplify their purchase process (Hoyer and Brown, 1990). In fact, this second option is quite usual as purchases decided in-store represent around 70% of total purchases (Stilley et al., 2010; Bell et al., 2011).

### Study data

We have combined three data sources in order to obtain information about computers offered for sale in one representative store in Spain of Europe's largest category killer computer retailer (Retail-Index, 2015): scanner data (to collect weekly data about units sold and prices), survey data (to collect assessment of perceived quality), and observational data (to capture the weeks in which displays or advertising flyers are used for promoting a particular SKU and for collecting from manufacturers' websites the quality attributed to intrinsic characteristics of the computers). Therefore, we created a whole database with several variables that indicate, for each SKU, its price and the number of weekly units sold, the assessment of its perceived quality by consumers depending on (i) its brand and (ii) its technical characteristics, and whether it was stimulated by in-store displays or featured on the advertising flyer each week.

#### Scanner data

We collected weekly sales data about computers offered for sale for a period of 8 weeks, from mid-February to mid-April 2011, in one representative store in Spain of Europe's largest category killer computer retailer (Retail-Index, 2015). We selected this short period of time in order to (i) avoid holiday periods (such as Christmas or Easter), which could cause any unusual peak or off-peak in this product's sales and (ii) prevent changes in certain issues relating to the analyzed commercial stimuli that could influence their effectiveness (such us the number of promoted SKUs or the placement where they are displayed/featured). The weekly data featured units sold and sales prices of 109 SKUs from all 12 brands offered by the retailer. The retailer provided us the information directly from the scanner data warehouse. This information included SKU stocks that were used to control whether any SKU was out-of-stock. If so, these weekly observations were deleted and not considered because its sales were zero since consumers could not purchase it. By contrast, we maintained observations without sales, if the SKU was offered at the point of sale, for our analysis. Furthermore, some SKUs that were offered for sale in the first weeks did not remain on sale during the whole 8-week study period because they were replaced by other more modern ones and, thus, other SKUs started to be offered by the store during the time period analyzed (e.g., third week, fourth week, etc.). These SKUs are considered in the database for the weeks in which there are offered for sale. Finally, we obtained 599 observations from 109 different SKUs. This information about prices and units sold is completed with other information sources, such as survey and observation, to detail the perceived quality and commercial stimuli.

#### Survey data

We conducted a consumer survey within the store over the same 8 weeks we collected the information about SKU prices and sales and the use of commercial stimuli. In this survey, we used different items to obtain (i) the assessment of perceived quality attributed to the brand for each of the 12 brands and (ii) the relative importance that different technical characteristics of computers have for the surveyed consumers. In fact, we used a five-point Likert scale for the assessment of a brand's perceived quality and we asked respondents to portion out 100 points among the technical characteristics according to their importance for the purchase. The items used in the questionnaire can be found in the Appendix Section (Appendix 1). First, we collected a pretest with 21 consumers and employees of the store in which we asked them to review the survey for any errors. After fixing a few minor mistakes, we randomly asked 402 customers who were observing and/or comparing computers inside the store, in fact, in the computer aisle or area. We decided to survey consumers who were interested on buying a computer, i.e., customers with a purchase intention, at the moment of making a decision because we believed their assessment could explain more reliably the influence of perceived quality and commercial stimuli on the computer sales. Finally, we obtained 376 valid surveys from consumers, 53 of whom considered themselves experts on computers.

#### Observational data

We used observational data for two objectives. On one hand, we visited the selected store once a week to capture when a display was used to stimulate purchase of a particular SKU and we reviewed the weekly advertising flyer each week to capture what computer products were featured in it. We also controlled for different issues that could influence the effect of these commercial stimuli, such as the number of items displayed or featured or the place within the store or in the advertising flyer in which the SKU was displayed or featured (Gijsbrechts et al., 2003; Bezawada et al., 2009; Chandon et al., 2009; Luceri et al., 2014). Thus, we preferred to select a shorter period of time in which any of these issues about commercial stimuli changed. For example, the number of displayed computers is the same for each week (except 1 week in which the number is four instead of five) and they were located at the same point within the store so that this issue would not have an influence on their effectiveness. Regarding weekly advertising flyers, they were launched in the same geographic area, with the same number of pages (eight), the same temporal frequency or duration (weekly), the same number of featured computer products (except 2 weeks in which the number is five instead of four) in the same pages and with average discounts without significant differences.

On the other hand, we checked the information about each computer product on the manufacturers' websites in order to obtain the assessment of quality attributed to intrinsic characteristics by its manufacturer. They usually use a five-star system in which they assess the quality of their computers' technical characteristics, on many occasions by following the assessment of the actual manufacturer of the components (e.g., Intel for processor). In the cases in which the manufacturer did not use this five-star system, we used the assessment from the manufacturer of components for the same technical attributes with the same characteristics (speed or capability) and we asked the retailer manager to give us his/her opinion or approval. The assessment used for each technical attribute can be found in the Appendix Section (Appendix 2). Table 2 provides some descriptive information of the three data sources that we collected and used in this study.

**Table 2:** **Descriptive study data**.

**Study Data**					
Time Period: mid-February—mid-April 2011 (8 weeks) Place: Salamanca (Spain)					
**Scanner data**		**Survey data**		**Observation data**	
Number of SKUs	109	Population	Computer buyers	Observations with displays	39
Number of brands	12	Pretest	21 persons	Observations with flyers	34
Number of observations	599	Total sample	376 persons		
		Sampling error to 95% confidence level	5%		

### Operationalization of variables

#### Measures of perceived quality

The customers' perceived product quality comprises both types of attributes, extrinsic and intrinsic, and therefore it was really necessary to measure these two dimensions independently: the perceived quality attributed to the brand and the perceived quality attributed to the intrinsic product characteristics. For these measures, we collected the consumer survey of potential buyers and paid attention to the technical characteristics of the computers.

For the “perceived quality attributed to the brand” (PQB) variable, we turned to assessments of the brands that consumers offered in the survey. Specifically, we directly asked about the level of quality they would attribute to each of the 12 brands in the panel data, on a five-point Likert scale following already validated quality scales, such as those from Keller and Aaker (1992), Grewal et al. (1998), and Erdem et al. (2006). This procedure provided us with a separate score for each of the 12 brands that were offered for sale in the study period. Therefore, we obtained a new enclosed variable—the values are between one and five—which collects the average score of the perceived quality attributed to the brand, according our surveyed consumers. As we searched and asked for the brand quality assessment, all products of a particular brand have the same value for this variable.

For the “perceived quality attributed to the intrinsic product characteristics” (PQIC) variable, we combined information related to the technical attributes and subjective information from the consumer survey. First, for each SKU, we sought the manufacturers' assessment of the four most important intrinsic technical characteristics for purchasing a computer (Mitra and Golder, 2006): processor speed, RAM capacity, hard drive capacity, and graphics card capability. Thus, we obtained enclosed values—between one and five—which collect the quality level of each these four technical characteristics depending on their objective features, such as capacity measured in *MegaBites/GigaBites* or speed measured in *GHz*. However, according to our surveyed consumers, these four intrinsic attributes do not have the same importance when they purchase a computer. Therefore, secondly, we weighted the scores obtained for quality level of these technical characteristics depending on their intrinsic features, by considering the average importance that our surveyed consumers granted these technical attributes. Thus, we obtained another enclosed variable—with values also between one and five—which collect the average score of the perceived quality attributed to the intrinsic characteristics for each of the 109 analyzed SKUs.

#### Measures of commercial stimuli

We collected the use of both analyzed commercial stimuli as two dummy variables, following previous studies (e.g., Narasimhan et al., 1996; Van Heerde et al., 2004; Inman et al., 2009). They take a value of 1 if the reference appears in the current week in the analyzed stimulus, and 0, otherwise.

For the displays variable, we obtained the observational information about their use by visiting the store during each of the weeks analyzed. During these visits, we explored the whole store and checked whether each SKU was located in a different placement than usual -such as an end-of-aisle or an island- and if it was stimulated by any special presentation or display.

Regarding the advertising flyer variable, we checked all the weekly advertising flyers that the retailer sent during the study period in order to create this dummy variable. This retailer usually launches a different advertising flyer every week by highlighting different SKUs from several product categories that it offers on sale. This advertising flyer is sent to potential consumers' home in a particular commercial area of influence of the store. Table 3 provides descriptions of the main variables used for the study.

**Table 3:** **Study variables**.

**Variable**		**Description**
*S_*it*_*	Sales	Physical units sold of product i on week t.
*PR_*it*_*	Price	Retail price in euros (including VAT) of product i on week t.
*BR_(*k*)*i*_*	Brands	Dummy variables that control for the effect of each brand. We create a dummy variable for each of the 12 brands, such that it takes a value of 1 if product i has the brand k. In fact, we introduce 11 dummy variables in the models because a brand is taken as a reference.
*DI_*it*_*	Display	Dummy variable, equal to 1 if product i is displayed with a special presentation or at a special location such as end-of-aisle or island on week t, and equal to 0 otherwise.
*AF_*it*_*	Advertising flyer	Dummy variable, equal to 1 if product i is featured on an advertising flyer or brochure on week t, and equal to 0 otherwise.
*PQB_*i*_*	Perceived quality attributed to the brand	The average assessment of the product quality attributed to the brand, according to surveyed consumers. The values vary between 1 and 5. All products of the same brand have the same value for this variable.
*PQIC_*i*_*	Perceived quality attributed to the intrinsic product characteristics	The weighting, according to the importance granted by surveyed consumers, of the assessment of the quality of product i depending on its main intrinsic technical characteristics. The possible values vary between 1 and 5.

i, is the SKU for which we collect information; t, is the week in which the information is collected; k, is the product brand

### Model and estimation

We proposed a sequential analysis, through multiple linear regressions, in which we considered the effect of each commercial stimulus and its interaction with both types of perceived quality separately in order to explain the weekly product sales. We then presented a general model that jointly compares the direct relationship of the perceived quality attributed to the brand and the perceived quality attributed to the intrinsic product characteristics with sales, as well as their moderating roles in the link between displays or advertising flyers and sales.

Then, we checked whether the necessary assumptions of normality, linearity and homoscedasticity were met. In addition, we found that there was no multicollinearity between the variables based on an analysis of tolerance and VIF (Hair et al., 1998).

Therefore, we defined different models for the sequential analysis. Model 1 is used to contrast the effectiveness of displays and advertising flyers for this product category, i.e., to contrast their direct effects on sales. Furthermore, the product price is used as control variable in order to analyze whether the price level is important in this product category as occurs in FMCG according to most previous studies. Note that we introduce 11 instead of 12 dummy variables for controlling the effect of each brand because a brand must be taken as a reference. Thus:

(1) $$\begin{array}{l} S_{it}=\alpha +\beta_{1}PR_{it}+\sum_{k=1}^{11}\beta_{2k}BR_{ki}+\beta_{3}DI_{it}+\beta_{4}AF_{it}+\varepsilon_{i} \end{array}$$

Where:

α: is the constant to be estimated

β_*j*_: are estimation parameters

ε: is the error

Moreover, Model 2 and Model 3 are used to analyze the moderating role of perceived quality on the effectiveness of displays and advertising flyers, respectively. Thus, Model 2 includes interactions of the variable that represents the use of displays in the store with the perceived quality attributed to the brand (DIxPQB_*it*_) and with the perceived quality attributed to the intrinsic product characteristics (DIxPQIC_*it*_). Model 3 features interactions between the variable that captures the use of advertising flyers and both types of perceived quality (AFxPQB_*it*_) and (AFxPQIC_*it*_). In both models, the direct effect of the perceived quality attributed to the brand gets omitted, due to its redundancy with the effects of brand dummy variables. Thus, the proposed models are expressed as follows:

(2) $$\begin{array}{l} S_{it}=\alpha +\beta_{1}PR_{it}+\sum_{k=1}^{11}\beta_{2k}BR_{ki}+\beta_{3}DI_{it}+\beta_{4}PQIC_{it}\\ \;+\;\beta_{5}DIxPQB_{it}+\beta_{6}DIxPQIC_{it}+\varepsilon_{i}, \end{array}$$

and

(3) $$\begin{array}{l} S_{it}=\alpha +\beta_{1}PR_{it}+\sum_{k=1}^{11}\beta_{2k}BR_{ki}+\beta_{3}AF_{it}+\beta_{4}PQIC_{it}\\ \;+\;\beta_{5}AFxPQB_{it}+\beta_{6}AFxPQIC_{it}+\varepsilon_{i} \end{array}$$

Finally, Model 4 is the general model that collects the direct effect of both commercial stimuli as well as their interactions with the perceived quality attributed to the brand and the perceived quality attributed to the intrinsic product characteristics on sales in order to obtain their moderating roles on the effectiveness of displays and advertising flyers to increase computer sales. Therefore, the general model is expressed as follows:

(4) $$\begin{array}{l} S_{it}=\alpha +\beta_{1}PR_{it}+\sum_{k=1}^{11}\beta_{2k}BR_{ki}+\beta_{3}DI_{it}+\beta_{4}AF_{it}\\ \;+\;\beta_{5}PQIC_{it}+\beta_{6}DIxPQB_{it}+\beta_{7}DIxPQIC_{it}\\ \;+\;\beta_{8}AFxPQB_{it}+\beta_{9}AFxPQIC_{it}+\varepsilon_{i} \end{array}$$

## Results

Results of the estimations are reported in Table 4. The results of Model 1 indicate that the use of both commercial stimuli, displays and advertising flyers, for promoting computer products have significant and positive direct effects on their sales (*p* < 0.01), in line with previous studies about frequently purchased products (Gijsbrechts et al., 2003; Van Heerde et al., 2004; Bezawada et al., 2009; Inman et al., 2009). These results confirm hypotheses H1 and H2. Moreover, we tested these results and the effects of both commercial stimuli are significantly different (*p* > 0.01), which highlights the need to analyze them separately. Furthermore, Model 1 already indicated that computer products have differences with respect to FMCG because it was observed that the effect of the price variable is not significant. One reason for this is that consumers may focus on the perceived quality attributed to the brand or the quality of the technical attributes in order to reduce the perceived risk linked to this complex product (Neelamegham and Chintagunta, 2004; Sriram et al., 2006). Accordingly, the perceived quality would be more important than prices for these infrequently purchased products.

**Table 4:** **Estimation results**.

**Variables**	**Model 1**	**Model 2**	**Model 3**	**Model 4**
Constant	0.414	−0.046	−0.350	−0.361
Price	0.001	−0.002	−0.002	−0.001
Acer	OUT^a^	OUT	OUT	OUT
Apple	−0.240	0.432	−0.361	−0.292
Asus	0.805	1.19^*^	0.222	0.340
Compaq	0.736	0.897	0.731	0.801
Dell	−1.029	−0.967	−1.258	−1.396^**^
HP	0.704	−0.395	1.514^*^	−0.429
LG	−0.782	−0.767	−0.729	−0.618
Medion	−0.231	0.399	0.191	0.239
PB	0.615	0.461	0.462	0.327
Samsung	1.823^**^	2.439^***^	1.625^*^	1.594^**^
Sony	4.081^***^	3.162^**^	5.303^***^	3.533^***^
Toshiba	5.144^***^	4.398^***^	6.229^***^	4.611^***^
Displays	8.197^***^	10.748^*^		5.933
Advertising Flyers	7.183^***^		3.800^*^	4.922
Perceived quality attributed to the brand		OUT^b^	OUT	OUT
Perceived quality attributed to intrinsic characteristics		0.633^*^	0.793^*^	0.600^*^
Displays–perceived quality attributed to the brand		5.037^***^		3.988^***^
Displays–perceived quality attributed to intrinsic characteristics		2.288^*^		2.149^*^
Advertising flyers–perceived quality attributed to the brand			1.327^*^	2.439^*^
Advertising flyers–perceived quality attributed to intrinsic characteristics			3.254^**^	4.303^**^
Adjusted R-square	0.429	0.393	0.331	0.472
F ANOVA	^***^	^***^	^***^	^***^

* *p < 0.1*;

** *p < 0.05*;

*** *p < 0.01*.

a *Acer is taken as the reference brand*.

b *The effect of Subjective Quality is redundant, because we introduce a constant for each brand*.

Model 2 indicates a significant positive moderating effect for both the perceived quality attributed to the brand (*p* < 0.01) and the perceived quality attributed to the intrinsic product characteristics (*p* < 0.10) on the effectiveness of displays to promote computer products. These results indicate that the higher the perceived quality of a product (both types of perceived quality), the greater the displays' effectiveness. It seems these findings are in line with the results of previous studies analyzing FMCG, such as Lemon and Nowlis (2002); however, they use price tiers as proxy of quality, and thus these findings are different and original. Furthermore, we distinguished between perceived quality attributed to the brand and perceived quality attributed to intrinsic attributes and the effects of both types of perceived quality are significantly different (*p* < 0.01). Thus, we may surmise that the effectiveness of displays on computer sales will be different depending on the two types of perceived quality.

Similarly, Model 3 indicates the moderating effect of both types of perceived quality on advertising flyers' effectiveness on computer sales. In fact, the interactions between advertising flyers and the perceived quality attributed to the brand (*p* < 0.10) and perceived quality attributed to the intrinsic product characteristics (*p* < 0.05) present a significant positive effect. The effect of these interactions are also significantly different (*p* < 0.01), indicating that each type of perceived quality has a different impact on the effectiveness of each analyzed commercial stimulus. As in the case of the displays, we may conjecture that the effectiveness of advertising flyers on computers sales will be different depending on the two types of perceived quality.

Finally, the results of Model 4 confirm that perceived quality attributed to the brand presents a greater moderating impact on displays (3.998, *p* < 0.01) than on advertising flyers (2.439, *p* < 0.10). These effects are significantly different (*p* < 0.01) and indicate that the perceived quality attributed to the brand enhances the effectiveness of displays more than it does the effectiveness of advertising flyers for sales; therefore, these results are in line with hypothesis H3. In addition, Model 4 also indicates that perceived quality attributed to the intrinsic product characteristics exerts a greater moderating impact on advertising flyers (4.303, *p* < 0.01) than on displays (2.149, *p* < 0.10). These effects are also significantly different (*p* < 0.01) and indicate that the perceived quality attributed to the intrinsic product characteristics enhances the effectiveness of advertising flyers more than it does the effectiveness of displays for sales; therefore these results are also in line with hypothesis H4. We summarize these results in Table 5.

**Table 5:** **Summary of results**.

**Hypotheses**	**Expected effect**	**Result**
H1	Product displays engender a positive effect on sales.	Confirmed
H2	Advertising flyers engender a positive effect on sales.	Confirmed
H3	The perceived quality attributed to the brand enhances the effectiveness of displays more than it does the effectiveness of advertising flyers for sales.	Confirmed
H4	The perceived quality attributed to the intrinsic product characteristics enhances the effectiveness of advertising flyers more than it does the effectiveness of displays for sales.	Confirmed

## Discussion

Product quality is a key determinant in the purchase of computer products, and in stimulating customer loyalty. Perceived quality has two dimensions, extrinsic quality—linked to the brand—and intrinsic quality—related to internal product characteristics. Whereas extrinsic attributes (brand name) are more related to affective loyalty (customers build affect toward the brand on the basis of cumulatively satisfying usage occasions), intrinsic attributes have a more objective nature and are more related to cognitive loyalty.

The main aim of this research was to analyze the importance of both perceived quality attributed to intrinsic characteristics and perceived quality attributed to the brand in explaining the effects of two commercial stimuli, displays inside the store where the customer has less time to process information and, advertising flyers sent to potential customers where the customer has more time to process information. We analyze an infrequently purchased product, computers, which feature substantial technological components and greater perceived risk, because of their complexity and dynamic evolution (Neelamegham and Chintagunta, 2004; Sriram et al., 2006).

According to our results, both stimuli, displays and advertising flyers, help increase sales in this product category. The positive influence on sales is moderated by the two dimensions of the products' perceived quality: one attributed to intrinsic characteristics (the product's own technical characteristics) and one attributed to the brand (assessment of brand quality). The more perceived quality a product has, the greater the impact of the displays and advertising flyers. However, we also emphasize that the perceived quality attributed to the brand improves to a greater extent the effect of displays, which work inside the store as a brand heuristic to help consumers with information processing. By contrast, we find that the perceived quality attributed to the intrinsic product characteristics improves to a greater extent the effect of advertising flyers, which are usually sent to potential consumers' home and favor systematic information processing.

These findings may be considered original and very useful for manufacturers and retailers because previous studies did not analyze the effectiveness of different commercial stimuli (one in-store and one out-of-store stimuli) on infrequently purchased products (such as computer products which feature substantial technological components and greater perceived risk, because of their complexity and dynamic evolution), and because we explain these results from the point of view of consumer behavior, focusing on the two dimensions of quality (perceived quality attributed to the brand and perceived quality attributed to intrinsic attributes). Therefore, our findings entail very important managerial implications for retailers as well as for manufacturers because they show what type of commercial stimuli is more appropriate for technological and infrequently purchased products depending on their extrinsic and intrinsic attributes.

Furthermore, these findings also have theoretical relevance in marketing, retail and consumer behavior research because they prove that commercial stimuli work differently depending on whether they take place in-store (time-constrained condition) or out-of-store (low time-constrained condition), since they trigger different types of purchasing processes (cognitive loyalty-intrinsic perceived quality or affective loyalty-extrinsic perceived quality). Because of this, unlike what was previously believed, commercial stimuli not only work for more hedonic or impulsive product purchases–which are usually acquired through an unplanned purchase process–but they may also be used for products with a higher perceived risk and product purchase involvement. To optimize sales performance, it is necessary to understand how consumers' perceived quality is formed by distinguishing between these two dimensions.

### Managerial guidelines for manufacturers and retailers

According to these results, we recommend that computer manufacturers allocate some resources to convincing retailers to encourage sales of their products through displays and advertising flyers; the results affirm their effectiveness for infrequently purchased products such as computers. In addition, they could devote further efforts to increase the perceived quality attributed to their brand, which may be more profitable in terms of enhancing their image than improving the intrinsic technical product characteristics. In this product category, consumers seek to reduce the high perceived risk associated with purchasing such an infrequently purchased, relatively expensive, complex product by focusing on the brand. Furthermore, many consumers also seek advice from other expert consumers or sellers, who have a great influence as prescribers. Thus, manufacturers should combine a *pull strategy* to attract potential buyers with a *push strategy* that encourages the retailer's sellers to recommend their brand instead of other brands.

For retailers, we suggest they pay close attention to their uses of displays and advertising flyers because both commercial stimuli are particularly effective in the computer product category. According to our results, retailers could use displays inside the store for products with higher perceived quality attributed to recognized brands in order to have consumers under time-constrained conditions use heuristics to simplify their decisions and make an unplanned purchase. In contrast, they could prominently feature products with higher intrinsic technical quality on their advertising flyers because consumers are likely to compare the product's attributes carefully and make more reasoned purchases under low time-constrained conditions.

Furthermore, buyers are not extremely price sensitive, perhaps because they assume price cuts when the product is displayed in a special location or featured on an advertising flyer (Gázquez-Abad et al., 2014). An additional reason could be that they focus on the perceived quality attributed to the brand or to the technical attributes for reducing the perceived risk linked to this complex product, rather than gain economic deals. In this line, the perceived quality would be more important than prices for these infrequently purchased products and retailers may optimize their profits by using commercial stimuli without needing to offer great price discounts.

### Limitations and further research

The major limitation of this study is that our data come from only one representative store in Spain belonging to Europe's largest category killer computer retailer. This was due to the need (i) to visit the store weekly in order to collect observational data about the use of displays, as well as (ii) to collect a consumer survey within the store in which the sales data were obtained about their product quality assessment. Related to this limitation, we only were able to use data on the SKUs and brands sold by only one store for a short period of time, although it does pertain to the most important retailer in Europe.

Therefore, given this limitation, further research could expand this study by collecting data from different stores and, even different retailer chains. It would be interesting to compare our results across (i) other category killers, (ii) other retail formats that also sell computers such as hypermarkets, or (iii) other geographic areas. Through these expansions, further research could examine if our findings are moderated by the retail chains' positioning and their characteristics (such as their assortment, their specialization level, their sales staff, etc.). Furthermore, it is possible that results may vary depending on behavioral loyalty, higher/lower price sensitivity, or the purchase involvement and perceived risk in this product category linked to the greater/lesser product knowledge level of consumers in other regions or countries.

Another important limitation is the use of a validated non-specific questionnaire for the assessment of subjective product quality and the importance of the four technical product attributes most analyzed for computer products. Thus, another interesting research extension might devise a specific questionnaire, adapted to technological products, to refine the measure of subjective quality.

In sum, to complement this study, further research might increase the number of SKUs and brands as well as the number of analyzed product attributes, for a longer study period, and examine our findings by considering different variables that could be moderating them, such as retailer positioning, retailer format or differences in behavioral variables depending on areas or countries, in order to gain further insights and improve the managerial guidelines that can be derived from the analysis.

## Author contributions

ÁG, ÓG, and MM have participated in the development of the conceptual framework and methodology as well as the data collection, the realization of the empirical analysis and the results.

### Conflict of interest statement

The authors declare that the research was conducted in the absence of any commercial or financial relationships that could be construed as a potential conflict of interest.

## Appendix 1. questionnaire on quality assessment of computer products

Dear client:

We are conducting a survey for a study by the University of Salamanca in the field of computer products.

If you want to contribute, you need only answer some brief questions according to your opinion. This will only take a few minutes. In order to ensure maximum confidentiality, your answers will be treated anonymously and in aggregate form.

Thank you very much for your willingness to complete this questionnaire.

### Questions

1. Distribute 100 points into these characteristics according their importance when you buy a computer.

**Table:**

**Technical atributtes**	**Points**
Processor speed (GHz)	
RAM capacity (Mb)	
Hard drive capacity (Mb)	
Graphics card capability (Mb)	
Total	100

2. Assess the quality of these brands according your opinion.

**Table:**

**Brand**	**Very low**	**Low**	**Regular**	**High**	**Very high**
Acer	1	2	3	4	5
Apple	1	2	3	4	5
Asus	1	2	3	4	5
Compaq	1	2	3	4	5
Dell	1	2	3	4	5
Hp	1	2	3	4	5
LG	1	2	3	4	5
Medion	1	2	3	4	5
PB	1	2	3	4	5
Samsung	1	2	3	4	5
Sony	1	2	3	4	5
Toshiba	1	2	3	4	5

## Appendix 2. assessments of technical attributes

**Table:**

**Processor**		**RAM**		**Hard drive**		**Graffic card**	
**Option**	**Score**	**Option**	**Score**	**Option**	**Score**	**Option**	**Score**
Celeron or ATOM processor	1	512 MB	1	≤249 GB	1	Integrated/Shared card	1
AMD, Pentium and ≤ P4400	2	1 GB	2	250–320 GB	2	256 MB dedicated	2
P4400-P6600 or i3 processor	3	2-3 GB	3	321–500 GB	3	512 MB dedicated	3
P6700-P8700 or i5 processor	4	4-5 GB	4	501–700 GB	4	1 GB dedicated	4
≥P8800 or i7 processor	5	≥6 GB	5	≥701 GB	5	≥2 GB dedicated	5
